# New technologies developed for treatment of premature ejaculation

**DOI:** 10.1038/s41443-024-00875-w

**Published:** 2024-03-27

**Authors:** Arik Shechter, Ilan Gruenwald

**Affiliations:** 1 Neuro-Urology Unit, Rambam Health Care Campus, Haifa, Israel; 2 Department of Family Medicine, Ruth and Bruce Rappaport Faculty of Medicine, Haifa, Israel; 3 The Bruce Rappaport Faculty of Medicine, the Technion—Israel Institute of Technology, Haifa, Israel; 4 Clalit Health Services, Haifa, Israel

**Keywords:** Therapeutics, Sexual dysfunction

## Abstract

Premature ejaculation (PE), lifelong and acquired, is the most common male sexual disorder, with serious impacts on the patient and his partner’s quality of life, sexual well-being, and psychosocial health. The most popular treatment options are on-demand topical anesthetics and off-label daily or on-demand selective serotonin reuptake inhibitors (SSRIs), followed by behavioral therapy. While SSRI treatments are reportedly safe, they are associated with limited efficacy and provide only a temporary delay in ejaculation latency time. The majority of PE patients are dissatisfied with SSRIs; thus, adherence to on-demand or daily SSRI treatments is low. In this article, we review studies on currently available technologies that are not pharmacological, surgical, cognitive or behavioral therapies. Recent data from studies of newly developed medical devices used in PE treatment are encouraging as they provide drug-free spontaneity during coitus, without severe adverse effects.

## Introduction

Premature ejaculation (PE), one of the most common male sexual disorders, profoundly affects the quality of life for both the patient and their partner [1]. Many of the proposed definitions for PE lack a foundation in scientific data and lack diagnostic criteria [2, 3]. The International Society for Sexual Medicine (ISSM) defines PE (lifelong and acquired) as characterized by the following criteria: ejaculation which almost always or always occurs prior to or within 1 min of vaginal penetration (lifelong PE) or a clinically significant and upsetting reduction in ejaculation latency time, of often up to 3 min (acquired PE); inability to delay ejaculation in nearly all or all vaginal penetrations (lifelong and acquired PE); and negative personal consequences, such as inconvenience, distress, frustration, and/or avoidance of sexual intimacy (lifelong and acquired PE) [4]. Nevertheless, this widely accepted definition by the ISSM is applicable to heterosexual penis-vagina sexual activities, while we have limited information on how to define PE in other sexual activities, such as anal sex or in men with homosexual orientations. Epidemiological studies, based on non-validated PE definitions and patient self-reported outcome (PRO) measures, found prevalence of PE complaints in the male population as high as 20–30% [5–9]. However, subsequent epidemiology studies applying evidence-based PE definitions found much lower prevalence rates (~5%) for both lifelong and acquired PE [10–13]. Of note, since PE is frequently a self-reported and self-rated complaint, it is difficult to determine its epidemiology. Complicating the matter further is the fact that PE is diagnosed in some couples based on distress and not on objective symptoms [14].

While the exact etiology of PE remains undetermined [15–18], the most widely accepted theories regarding the etiology of lifelong PE center on disruptions in neurotransmitter activities within the central nervous system. This includes serotonin, noradrenaline, oxytocin, nitric oxide (NO), and Gama aminobutyric acid (GABA) [19]. Additionally, increased sensitivity of the glans penis [20], erectile dysfunction (ED) [21, 22], genetic polymorphisms [23–26], hormonal imbalances [27, 28], and prostatic diseases [29, 30] also contribute to its pathophysiology. Further research is necessary to evaluate the impact of these factors on ejaculation physiology. The etiology of acquired PE is more closely associated with underlying medical, psychological, and interpersonal causes, as described by Serefoglu et al. [31].

Since the 1990s, the prevailing treatment options for both lifelong and acquired PE have been on-demand topical anesthetics and off-label daily or on-demand selective serotonin reuptake inhibitors (SSRIs) [32], including Dapoxetine, a swiftly absorbed and short-acting SSRI, which stands as the sole approved oral medication for PE treatment. Notably, Dapoxetine lacks approval from the US Food and Drug Administration due to its uncertain efficacy and safety [32]. While the majority of men with PE report these treatments as safe, some may experience minor adverse effects. Additionally, SSRIs offer only a temporary delay in ejaculation latency time, with PE often resurfacing after treatment cessation [32–36]. Certain PE patients and their partners might find the necessity to apply a topical anesthetic 5–10 min before each sexual encounter dissatisfying [37]. A post-marketing study reveals that a significant majority (up to 75%) of PE patients express dissatisfaction with topical anesthetic treatments. Consequently, adherence to topical anesthetic treatments remains low, at ~10% [38].

Behavioral therapy, specifically sex therapy, represents a treatment with fewer side effects and lower costs. Its goal is to enhance self-confidence and alleviate anxiety and depression by systematically training men to acquire sexual skills that can extend ejaculation time. In the short term, behavioral therapy can yield success rates ranging from 45% to 65% of patients, but its long-term effects remain uncertain [39].

A definitive cure for PE is still elusive, and ongoing research is focused on identifying the optimal treatment for this condition. Consequently, in response to the unmet need for PE therapy, new technologies are currently under development.

This review article is aimed at providing a summary of the presently available non-pharmacological, non-surgical technological therapies for PE, excluding cognitive or behavioral approaches. We will discuss the findings from studies that are dedicated to creating innovative technological treatment options for PE.

### Neuromuscular electrical stimulation

Pelvic floor muscles (PFMs), specifically the ischiocavernosus and bulbospongiosus muscles, assume a pivotal role in the expulsion phase of ejaculation, expressed by increase in electromyographic activity during ejaculation [40].

The objective of physio-kinesiotherapy and electrostimulation is to augment the contractile strength of the perineal muscles, complemented by biofeedback to facilitate patients in mastering the recognition and contraction of PFMs, thereby strengthening the urethral sphincter. However, a comprehensive understanding of the intricate protocol dynamics is often necessitated, requiring patients to undergo several months of PFM training to gain control over the ejaculatory reflex and adeptly apply acquired skills during sexual activity [40].

In a study by Pastore et al. [41], 40 patients with lifelong PE and intravaginal ejaculatory latency time (IELT) values below 1 min underwent a 12-week PFM rehabilitation regimen, comprising physio-kinesiotherapy, trans anal probe electro-stimulation, and thrice-weekly biofeedback sessions. Post-intervention, the mean IELT demonstrated a significant increase compared to baseline values (31.7 s vs. 146.2 s, respectively, *P* < 0.0001). Subsequently, Pastore et al. [42] retrospectively reviewed 154 participants with baseline IELT values of 60 s or less and Premature Ejaculation Diagnostic Tool (PEDT) scores exceeding 11. The 12-week PFM rehabilitation program included physio-kinesiotherapy, trans anal probe electrostimulation, and three weekly biofeedback sessions, each lasting 20 min. Of the 122 participants completing PFM rehabilitation, 111 gained control over their ejaculation reflex, resulting in a mean IELT of 161.6 s and a PEDT score of 2.3 at the intervention endpoint, indicating a significant increase from baseline IELT of 40.4 s and PEDT score of 17.0 (*P* < 0.0001). At the 36-month follow-up, 64% and 56% of the remaining 95 participants maintained satisfactory ejaculation control at 24- and 36-months post-intervention, respectively.

Protocols utilizing PFM rehabilitation, incorporating physio-kinesiotherapy, trans-anal probe electrostimulation, and biofeedback, are characterized as protracted and cumbersome, lacking on-demand suitability during intercourse. Patients often necessitate substantial time to comprehend the intricacies of the protocol, essential for achieving control over their ejaculatory reflex and subsequently applying this knowledge during sexual activity.

Gruenwald et al. [43] proposed an alternative approach to PE treatment, utilizing transcutaneous electrical neuro stimulation (TENS) on the perineal region. The rationale was that TENS would suppress rhythmic contractions in the expulsion phase by generating a plateau action potential, through continuous stimulation of the bulbospongiosus and ischiocavernosus muscles. Anticipated benefits included hindering muscle relaxation, sustaining the muscles in a sub-tetanic contraction state, thus potentially leading to delayed ejaculation during sexual intercourse.

In 2017, Shechter et al. [44] piloted a study to test this hypothesis, employing a commercial TENS device on the perineum of 23 patients with lifelong PE, with each patient serving as their own control. The study compared Masturbating Ejaculatory Latency Time (MELT) with and without TENS during self-sexual stimulation. Results indicated significantly higher mean MELT values during TENS treatment compared to self-stimulation without TENS (311.4 s vs. 124.6 s, *P* = 0.0009), signifying an ~4-fold increase in MELT. Notably, the absence of an established MELT threshold in the literature prompted the researchers to assume a correlation with IELT in PE patients, an assumption lacking scientific validation and constituting a significant study limitation. No patients reported erectile difficulties or severe adverse effects, although a minority experienced minor adverse effects such as discomfort during stimulation and dysuria.

This study presents a novel approach to addressing lifelong PE through the extension of on-demand coital duration, achieved via electric stimulation of the ejaculation muscles using the In2 patch. This method holds promise as a potential on-demand, non-invasive, and drug-free treatment for PE. However, it is important to note that the study’s scope was constrained by a limited number of participants, the exclusion of those with acquired PE, short-term follow-up, exclusive focus on vaginal penetration, omission of men engaged in anal penetration, exclusion of couples with shorter-term relations, and the use of a device based on a theoretical mechanism of action.

As a result of this study, the In2 patch, a miniaturized on-demand perineal TENS device, was developed for PE treatment (Virility Medical Ltd., Hod Hasharon, Israel). During 2019–2020, Shechter et al. [45] conducted an international, bi-center, prospective, double-blind, randomized, bi-arm, sham-controlled, first-in-human clinical study to evaluate the safety, feasibility, and effectiveness of the perineal TENS device during coitus.

The study enrolled 59 male patients with lifelong PE, averaging 39.8 years old. IELT was measured by their female partner over a 2-week run-in period, and eligibility was determined based on IELT values and medical/sexual history. Patient-specific sensory and motor activation thresholds during perineal stimulation with the In2 patch were derived from these results. At the final visit, IELTs, Clinical Global Impression of Change (CGIC) scores, and Premature Ejaculation Profile (PEP) questionnaire outcomes were recorded. Each individual was compared to his own results, with and without the device, and comparison was also performed between the Sham and the Active groups. The primary end points evaluated the device’s efficacy as the mean change in geometric mean IELT.

51 of 59 patients completed the study. Of those, 34 were in the Active Group, and 17 were in the Sham Group. The baseline geometric mean IELT significantly increased from 67 to 123 s (*p* < 0.01) in the Active Group, compared to an insignificant increase from 63 to 81 s (*P* = 0.17) in the Sham Group. The mean increase in IELT from baseline was 56 s in the active Group, which was significantly different from, and 3.1 times greater, than the geometric mean increase of 18 s in sham group (*p* = 0.01), while the geometric mean fold increases in IELTs were 1.7 and 1.2 for Active and Sham groups, respectively. The mean ratio of fold change (Active/Sham) was 1.4, significantly different from 1.0 (*P* = 0.02).

In a subgroup analysis of subjects exhibiting improvement in IELT, 91% (31/34) of individuals in the Active Group demonstrated enhanced IELT during the treatment period compared to baseline. Responders exhibited a mean time-fold increase in IELT of 2.04, with a 95% confidence interval ranging from 1.68 to 2.40.

No instances of serious or severe treatment-emergent adverse events (AEs) were reported. Two minor AEs occurred in the Active Group (2/186 sessions), both attributed to the study device. One participant reported “Discomfort due to device vibration in the inguinal scar site,” while another reported “Pain and discomfort during sexual intercourse in the pelvic area”; nonetheless, both individuals continued their participation in the study. Consequently, the AE rate was 1.1% in the Active Group, contrasting with 0.0% (0/70) in the Sham Group (*P* = 1.00).

This study introduces an innovative approach to addressing lifelong PE by extending on-demand coital duration through electric stimulation of the ejaculation muscles using the In2 patch. This method shows promise as a potential on-demand, non-invasive, and drug-free treatment for PE. However, it is essential to acknowledge the study’s limitations, including a restricted participant pool, exclusion of individuals with acquired PE, short-term follow-up, a focus solely on vaginal penetration, omission of men engaged in anal penetration, exclusion of couples with shorter-term relations, and utilization of a device based on a theoretical mechanism of action. Future comparative studies are imperative to ascertain whether on-demand TENS treatment methods can match or surpass the efficacy of pharmacological agents in delaying ejaculation for patients with PE. The concept of utilizing electrical stimulation to prolong ejaculation latency time gained support from Cizmezi et al. in an animal model. Twenty-four male Wistar albino rats underwent neuromuscular electrical stimulation at the bulbospongiosus muscle and they measured the effect on ejaculation parameters. In this controlled study, high-frequency burst and continuous low-frequency (LF) neuromuscular electrical stimulation was applied to the rats for 30 min (n = 8 for each group including control). They found a significant difference between the groups in terms of ejaculation time (1344.71 ± 105.9, *p* = 0.002). Other measured PE parameters did not differ significantly between the groups (change in basal seminal vesicle pressure, seminal vesicle maximum pressure, number and interval time of seminal vesicle contractions and bulbospongiosus muscle EMG activities). They concluded that continues low-frequency neuromuscular electrical stimulation (2 Hz and 200 µs transition time) significantly prolonged the ejaculation time in rats. This study strengthens the theoretical mode of action by maintaining sub-tetanic continuous contraction that prevents the rhythmic contractions necessary for completing the ejaculatory process [46].

### Transcutaneous posterior tibial nerve stimulation (TPTNS)

TPTNS therapy has found extensive application in pelvic floor physiotherapy [47]. The underlying principle of electrostimulation therapy is rooted in the intricate sensorimotor function of the posterior tibial nerve, originating from T4–S3 roots. While the emission phase of ejaculation is primarily governed by stimuli from the T12–L1 area [48], the expulsion phase is predominantly regulated at the S2–S4 level [49, 50]. Consequently, TPTNS has the potential to inhibit both the emission (through the sympathetic system) and expulsion (through the parasympathetic–somatic ejaculation system) phases of ejaculation.

In a phase II trial, TPTNS was assessed as a novel treatment approach for PE. Eleven patients with PE underwent TPTNS sessions lasting 30 min, three times a week for 12 weeks. In total, 6 out of 11 (54%) patients who completed the 12-week treatment period exhibited a three-fold increase in IELT compared to baseline (*P* = 0.037). Only two patients reported complications, such as constipation (n = 1) and a sensation of heat in the leg (n = 1). Importantly, no reported adverse effects led to a change in therapy adherence. However, it is important to note that this study has significant limitations, including a very low number of participants, the absence of a control group, and a lack of randomization [51]. In a trial conducted in 2020, the effectiveness of TPTNS treatment was compared to sham therapy in a group of 60 men with PE. They underwent 30-min sessions of either TPTNS or sham therapy once a week. At the conclusion of the 12-week treatment period, the average IELT values increased from 40.4 s to 51.25 s for patients receiving TPTNS, while for those treated with sham therapy, the values went from 37.9 s to 42.5 s (*P* = 0.030) [52]. This study revealed an improvement in IELT scores in the Sham group, even though no electrical current was applied. This suggests that the contact of the TPTNS probe with the body may have induced a placebo effect. There were no statistically significant differences observed between patients treated with TPTNS or Sham in terms of the percentage change in IELT scores from pre-to-post-procedure (0.38 ± 0.47 vs. 0.23 ± 0.67, *P* = 0.415). However, it’s important to note that a major limitation of this study was the absence of randomization, potentially introducing bias in participant selection. The initial outcomes of TPTNS in treating PE appear conflicting, highlighting the need for randomized controlled studies involving large patient cohorts.

### Masturbation aid device

In recent years, a new approach to treating PE has emerged, involving the use of a masturbation aid device in conjunction with behavioral techniques. In a multicenter randomized clinical trial utilizing a parallel group design to assess the effectiveness of the electronic masturbation device known as Myhixel © (MYHIXEL, Seville, Spain) in PE treatment, Rodriguez et al. assigned 52 patients to two treatment groups, with only 40 completing the study. All patients underwent an 8-week Sphincter Control Training program. The sole distinction between the groups was the inclusion of the Myhixel © device. The primary metric was the “fold increase” in IELT. At the conclusion of the 8-week treatment, the geometric means of IELT demonstrated more favorable outcomes for the device group, albeit without statistical significance (*P* = 0.11) (an increase of 30 s versus 90 s from baseline in the exercise-only and device groups, respectively). Notably, in the device group, the fold increase in IELT was significantly higher compared to the exercise-only group, at 4.27 versus 2.09, respectively (*P* = 0.001) [53] (See Table 1 for comparison between the new technologies).

**Table 1:** Comparison between new scientifically studied technologies for the possible treatment of premature ejaculation.

#### Devices scientifically unproven for PE treatment

While there are treatments for PE that are actually, clinically proven, scientifically significant and FDA cleared (i.e behavioral therapies, topical anesthetics, and certain medications and medical devices), there are also numerous devices and products on the market that claim to treat or prevent PE but lack scientific evidence to support their efficacy (i.e Morari device- https://www.morarimedical.com/ #how-it-works, Vaacum constriction devices, male vibrators). It’s crucial to approach any device claiming to treat PE with skepticism and caution unless there is robust scientific evidence supporting its efficacy and safety.

## Conclusions

Currently, the standards of care in PE treatment are mainly SSRIs and topical anesthetics. However, the available drug treatments for PE come with limited effectiveness, a range of adverse effects, and a high rate of discontinuation. A definitive cure for PE, or at least therapies allowing for spontaneity during intercourse, remain elusive. Encouragingly, data from studies utilizing newly developed technological techniques and medical devices in PE treatment show promise. Solutions that are drug-free, entail minimal adverse effects, and permit spontaneity during intercourse are on the horizon. However, additional clinical studies would be beneficial in confirming the effectiveness of these therapies, potentially establishing them as possible alternatives to pharmacological PE treatments.

## Data Availability

This is a review article with no original scientific data.

## References

[1] Althof S. Prevalence, characteristics and implications of premature ejaculation/rapid ejaculation. J Urol. 2006;175:842–8. 16469562 10.1016/S0022-5347(05)00341-1

[2] American Psychiatric Association. Diagnostic and statistical manual of mental disorders: DSM-5. 5th ed. American Psychiatric Association, Washington, DC, 2013.

[3] Parnham A, Serefoglu EC. Classification and definition of premature ejaculation. Transl Androl Urol. 2016;5:416–23. 27652214 10.21037/tau.2016.05.16 PMC5001991

[4] Serefoglu EC, McMahon CG, Waldinger MD, Althof SE, Shindel A, Adaikan G, et al. An evidence-based unified definition of lifelong and acquired premature ejaculation: report of the second International Society for Sexual Medicine AdHoc Committee for the definition of premature ejaculation. Sex Med. 2014;2:41–59. 25356301 10.1002/sm2.27 PMC4184676

[5] Porst H, Montorsi F, Rosen RC, Gaynor L, Grupe S, Alexander J. The premature ejaculation prevalence and attitudes (PEPA) survey: prevalence, comorbidities, and professional help-seeking. Eur Urol. 2007;51:816–23. 16934919 10.1016/j.eururo.2006.07.004

[6] Laumann EO, Paik A, Rosen RC. Sexual dysfunction in the United States: prevalence and predictors. JAMA. 1999;281:537–44. 10022110 10.1001/jama.281.6.537

[7] Tang WS, Khoo EM. Prevalence and correlates of premature ejaculation in a primary care setting: a preliminary cross-sectional study. J Sex Med. 2011;8:2071–8. 21492404 10.1111/j.1743-6109.2011.02280.x

[8] McMahon CG, Lee G, Park JK, Adaikan PG. Premature ejaculation and erectile dysfunction prevalence and attitudes in the Asia-Pacific region. J Sex Med. 2012;9:454–65. 22023395 10.1111/j.1743-6109.2011.02507.x

[9] Saitz TR, Serefoglu EC. The epidemiology of premature ejaculation. Transl Androl Urol. 2016;5:409–15. 27652213 10.21037/tau.2016.05.11 PMC5001986

[10] Serefoglu EC, Yaman O, Cayan S, Asci R, Orhan I, Usta MF, et al. Prevalence of the complaint of ejaculating prematurely and the four premature ejaculation syndromes: results from the Turkish Society of Andrology Sexual Health Survey. J Sex Med. 2011;8:540–8. 21054799 10.1111/j.1743-6109.2010.02095.x

[11] Althof SE, McMahon CG, Waldinger MD, Serefoglu EC, Shindel AW, Adaikan PG, et al. An update of the International Society of Sexual Medicine’s Guidelines for the diagnosis and treatment of premature ejaculation (PE). Sex Med. 2014;2:60–90. 25356302 10.1002/sm2.28 PMC4184677

[12] Waldinger MD, Quinn P, Dilleen M, Mundayat R, Schweitzer DH, Boolell M. A multinational population survey of intravaginal ejaculation latency time. J Sex Med. 2005;2:492–7. 16422843 10.1111/j.1743-6109.2005.00070.x

[13] Waldinger MD, McIntosh J, Schweitzer DH. A five-nation survey to assess the distribution of the intravaginal ejaculatory latency time among the general male population. J Sex Med. 2009;6:2888–95. 19627471 10.1111/j.1743-6109.2009.01392.x

[14] Jannini EA, Lenzi A. Epidemiology of premature ejaculation. Curr Opin Urol. 2005;15:399–403. 16205491 10.1097/01.mou.0000182327.79572.fd

[15] Gopalakrishna A, Bole R, Alom M, Meng Y, Jimbo M, Trost L, et al. Characteristics of men who are bothered by rapid ejaculation: results from clinical intake surveys. Int J Impot Res. 2021;33:369–75. 32332929 10.1038/s41443-020-0277-x

[16] Saitz TR, Serefoglu EC. Advances in understanding and treating premature ejaculation. Nat Rev Urol. 2015;12:629–40. 26502991 10.1038/nrurol.2015.252

[17] Culha MG, Tuken M, Gonultas S, Cakir OO, Serefoglu EC. Frequency of etiological factors among patients with acquired premature ejaculation: prospective, observational, single-center study. Int J Impot Res. 2020;32:352–7. 31477853 10.1038/s41443-019-0188-x

[18] McMahon CG, Jannini EA, Serefoglu EC, Hellstrom WJ. The pathophysiology of acquired premature ejaculation. Transl Androl Urol. 2016;5:434–49. 27652216 10.21037/tau.2016.07.06 PMC5001985

[19] Waldinger MD. The pathophysiology of lifelong premature ejaculation. Transl Androl Urol. 2016;5:424–33. 27652215 10.21037/tau.2016.06.04 PMC5001987

[20] Donatucci CF. Etiology of ejaculation and pathophysiology of premature ejaculation. J Sex Med. 2006;3:303–8. 16939474 10.1111/j.1743-6109.2006.00305.x

[21] Guo L, Liu Y, Wang X, Yuan M, Yu Y, Zhang X, et al. Significance of penile hypersensitivity in premature ejaculation. Sci Rep. 2017;7:10441. 28874780 10.1038/s41598-017-09155-8 PMC5585329

[22] Corona G, Rastrelli G, Limoncin E, Sforza A, Jannini EA, Maggi M. Interplay between premature ejaculation and erectile dysfunction: a systematic review and meta-analysis. J Sex Med. 2015;12:2291–300. 26552599 10.1111/jsm.13041

[23] Fu X, Zhang X, Jiang T, Huang Y, Cheng P, Tang D, et al. Association between lifelong premature ejaculation and polymorphism of tryptophan hydroxylase 2 gene in the Han population. Sex Med. 2020;8:223–9. 32169437 10.1016/j.esxm.2020.02.004 PMC7261684

[24] Janssen PK, Schaik R, Olivier B, Waldinger MD. The 5-HT2C receptor gene Cys23Ser polymorphism influences the intravaginal ejaculation latency time in Dutch Caucasian men with lifelong premature ejaculation. Asian J Androl. 2014;16:607–10. 24799636 10.4103/1008-682X.126371 PMC4104091

[25] Salem AM, Kamel II, Rashed LA, GamalEl Din SF. Effects of paroxetine on intravaginal ejaculatory latency time in Egyptian patients with lifelong premature ejaculation as a function of serotonin transporter polymorphism. Int J Impot Res. 2017;29:7–11. 27679962 10.1038/ijir.2016.36

[26] Eltonsi TK, Tawfik TM, Rashed LA, GamalEl Din SF, Mahmoud MA. Study of the link between dopamine transporter gene polymorphisms and response to paroxetine and escitalopram in patients with lifelong premature ejaculation. Int J Impot Res. 2017;29:235–9. 28904397 10.1038/ijir.2017.29

[27] Abu El-Hamd M, Farah A. Possible role of serum testosterone, gonadotropins and prolactin in patients with premature ejaculation. Andrologia. 2018; 10.1111/and.12808. 10.1111/and.12808 28439908

[28] Cihan A, Demir O, Demir T, Aslan G, Comlekci A, Esen A. The relationship between premature ejaculation and hyperthyroidism. J Urol. 2009;181:1273–80. 19185321 10.1016/j.juro.2008.10.150

[29] Sihotang RC, Alvonico T, Taher A, Birowo P, Rasyid N, Atmoko W. Premature ejaculation in patients with lower urinary tract symptoms: a systematic review. Int J Impot Res. 2021;33:516–24. 32393845 10.1038/s41443-020-0298-5

[30] Shamloul R, el-Nashaar A. Chronic prostatitis in premature ejaculation: a cohort study in 153 men. J Sex Med. 2006;3:150–4. 16409229 10.1111/j.1743-6109.2005.00107.x

[31] Serefoglu EC, Cimen HI, Atmaca AF, Balbay MD. The distribution of patients who seek treatment for the complaint of ejaculating prematurely according to the four premature ejaculation syndromes. J Sex Med. 2010;7:810–5. 19912501 10.1111/j.1743-6109.2009.01570.x

[32] Mondaini N, Fusco F, Cai T, Benemei S, Mirone V, Bartoletti R. Dapoxetine treatment in patients with lifelong premature ejaculation: the reasons of a “Waterloo”. Urology. 2013;82:620–4. 23987156 10.1016/j.urology.2013.05.018

[33] Waldinger MD, Zwinderman AH, Schweitzer DH, Olivier B. Relevance of methodological design for the interpretation of efficacy of drug treatment of premature ejaculation: a systematic review and meta-analysis. Int J Impot Res. 2004;16:369–81. 14961051 10.1038/sj.ijir.3901172

[34] Waldinger MD. Towards evidence-based drug treatment research on premature ejaculation: a critical evaluation of methodology. Int J Impot Res. 2003;15:309–13. 14562129 10.1038/sj.ijir.3901023

[35] McMahon CG, Porst H. Oral agents for the treatment of premature ejaculation: review of efficacy and safety in the context of the recent International Society for Sexual Medicine criteria for lifelong premature ejaculation. J Sex Med. 2011;8:2707–25. 21771283 10.1111/j.1743-6109.2011.02386.x

[36] Salonia A, Rocchini L, Sacca’ A, Pellucchi F, Ferrari M, Del Carro U, et al. Acceptance of and discontinuation rate from paroxetine treatment in patients with lifelong premature ejaculation. J Sex Med. 2009;6:2868–77. 19656274 10.1111/j.1743-6109.2009.01404.x

[37] Boeri L, Pozzi E, Fallara G, Montorsi F, Salonia A. Real-life use of the eutectic mixture lidocaine/prilocaine spray in men with premature ejaculation. Int J Impot Res. 2022;34:289–94. 33828264 10.1038/s41443-021-00424-9

[38] Abu El-Hamd M. Effectiveness and tolerability of lidocaine 5% spray in the treatment of lifelong premature ejaculation patients: a randomized single-blind placebo-controlled clinical trial. Int J Impot Res. 2021;33:96–101. 31896832 10.1038/s41443-019-0225-9

[39] Bao B, Shang J, Wang J, Dai H, Li X, Zhang K, et al. Efficacy and safety of behavioral therapy for premature ejaculation: protocol for a systematic review. Medicine. 2019;98:e14056. 30653115 10.1097/MD.0000000000014056 PMC6370165

[40] Pischedda A, Fusco F, Curreli A, Grimaldi G, Farina FP. Pelvic floor and sexual male dysfunction. Arch Ital Urol Nefrol Androl. 2013;85:1–7. 10.4081/aiua.2013.1.7 23695397

[41] Pastore AL, Palleschi G, Fuschi A, Maggioni C, Rago R, Zucchi A, et al. Pelvic floor muscle rehabilitation for patients with lifelong premature ejaculation: a novel therapeutic approach. Ther Adv Urol. 2014;6:83–8. 24883105 10.1177/1756287214523329 PMC4003840

[42] Pastore, AL, Palleschi G, Fuschi A, Al Salhi Y, Zucchi A, et al. Pelvic muscle floor rehabilitation as a therapeutic option in lifelong premature ejaculation: long-term outcomes. Asian J Androl. 2018;20:572–5. 29974885 10.4103/aja.aja_30_18 PMC6219291

[43] Gruenwald I, Serefoglu EC, Gollan T, Springer S, Meiry G, Appel B, et al. Transcutaneous neuromuscular electrical stimulation may be beneficial in the treatment of premature ejaculation. Med Hypotheses. 2017;109:181–3. 29150283 10.1016/j.mehy.2017.10.008

[44] Shechter A, Serefoglu EC, Gollan T, Springer S, Meiry G, Appel B, et al. Transcutaneous functional electrical stimulation — a novel therapy for premature ejaculation: results of a proof of concept study. Int J Impot Res. 2020;32:440–5. 31570825 10.1038/s41443-019-0207-y

[45] Shechter A, Mondaini N, Serefoglu EC, Gollan T, Appel B, Gruenwald I. A Novel, on-demand therapy for Lifelong Premature Ejaculation using a Miniature Trans-Perineal Electrical Stimulator, the vPatch® - as-treated analysis. J Sex Med. 2023;20:22–9. 36897239 10.1093/jsxmed/qdac012

[46] Cizmeci S, Ongun S, Sarac A, Sel E, Tuzburun S and Durmus N. Low frequency neuromuscular electrical stimulation applied to the bulbospongiosus muscle prolongs the ejaculation latency in a rat model. Int J Impot Res. 2023; 10.1038/s41443-023-00678-5 10.1038/s41443-023-00678-5 36782022

[47] Peyronnet B, Amarenco G, Kerdraon J, Cornu JN, Gamé X. Transcutaneous posterior tibial nerve stimulation: ready for prime time. Neurourol Urodyn. 2019;38:1024–5. 30742341 10.1002/nau.23932

[48] Polat Dunya C, Tulek Z, Kürtüncü M, Panicker JN, Eraksoy M. Effectiveness of the transcutaneous tibial nerve stimulation and pelvic floor muscle training with biofeedback in women with multiple sclerosis for the management of overactive bladder. Mult Scler. 2021;27:621–9. 32513049 10.1177/1352458520926666

[49] Giuliano F. Neurophysiology of erection and ejaculation. J Sex Med. 2011;8:310–5. 21967393 10.1111/j.1743-6109.2011.02450.x

[50] Puppo V, Puppo G. Comprehensive review of the anatomy and physiology of male ejaculation: premature ejaculation is not a disease. Clin Anat. 2016;29:111–9. 26457680 10.1002/ca.22655

[51] Uribe OL, Sandoval-Salinas C, Corredor HA, Martínez JM, Saffon JP. Transcutaneous electric nerve stimulation to treat patients with premature ejaculation: phase II clinical trial. Int J Impot Res. 2020;32:434–9. 31551577 10.1038/s41443-019-0196-x

[52] Aydos MM, Nas I, Önen E. The impact of transcutaneous posterior tibial nerve stimulation in patients with premature ejaculation. Eur Res J. 2020;6:457–63.

[53] Rodríguez JE, Picazo JA, Marzo JC, Piqueras JA, Reina L, Hidalgo G, et al. Efficacy of Sphincter Control Training and medical device in the treatment of premature ejaculation: a multicenter randomized controlled clinical trial. PLoS One. 2021;16:e0257284. 34547013 10.1371/journal.pone.0257284 PMC8454929

